# Analysis of the Seismic Performance of Isolated Buildings according to Life-Cycle Cost

**DOI:** 10.1155/2015/495042

**Published:** 2015-01-05

**Authors:** Yu Dang, Jian-ping Han, Yong-tao Li

**Affiliations:** ^1^Key Laboratory of Concrete and Prestressed Concrete Structure, Ministry of Education, Nanjing 210096, China; ^2^School of Civil Engineering, Lanzhou University of Technology, Lanzhou, Gansu 730050, China

## Abstract

This paper proposes an indicator of seismic performance based on life-cycle cost of a building. It is expressed as a ratio of lifetime damage loss to life-cycle cost and determines the seismic performance of isolated buildings. Major factors are considered, including uncertainty in hazard demand and structural capacity, initial costs, and expected loss during earthquakes. Thus, a high indicator value indicates poor building seismic performance. Moreover, random vibration analysis is conducted to measure structural reliability and evaluate the expected loss and life-cycle cost of isolated buildings. The expected loss of an actual, seven-story isolated hospital building is only 37% of that of a fixed-base building. Furthermore, the indicator of the structural seismic performance of the isolated building is much lower in value than that of the structural seismic performance of the fixed-base building. Therefore, isolated buildings are safer and less risky than fixed-base buildings. The indicator based on life-cycle cost assists owners and engineers in making investment decisions in consideration of structural design, construction, and expected loss. It also helps optimize the balance between building reliability and building investment.

## 1. Introduction

The life-cycle cost of a building is the entire cost over its expected life time, including initial investment, maintenance, and repair costs. It also covers loss in occasional cases, such as that incurred during earthquakes. The optimum seismic performance of a building can be considered “the reasonable balance between the initial investment cost of improving seismic performance and the prospective loss as a result of earthquakes” [1]. Life-cycle cost can be regarded as an indicator of structural seismic performance because the cost of earthquake damage can be quantified.

Many of the following studies determine the optimal seismic design by minimizing life-cycle cost. Nathwani et al. [2], Pandey and Nathwani [3], and Rackwitz [4] developed optimal designs simply by minimizing the expected life-cycle cost based on its magnitude of uncertainty. Liu et al. [5] suggested a two-objective optimization procedure to design steel moment-resisting frame buildings within a performance-based seismic design framework. In this procedure, the initial material and life time costs of seismic damage are treated as two separate objectives. Lagaros et al. [1] adopted the limit-state cost to compare descriptive and performance-based design procedures. Frangopol and Liu [6] reviewed the recent developments in life-cycle maintenance and management planning for deteriorating civil infrastructures, especially bridges. Kappos and Dimitrakopoulos [7] implemented decision-making tools, namely, cost-benefit and life-cycle cost analyses, to determine the feasibility of strengthening reinforced-concrete buildings. Pei and van de Lindt [8] also proposed a probabilistic framework to estimate long-term, earthquake-induced economic loss related to wood-frame structures.

Several studies have analyzed the life-cycle cost of isolated buildings. Lee et al. [9] studied the life-cycle cost of a structure with base isolation. The results of life-cycle cost analysis indicate that isolators reduced the life-cycle cost by approximately 16%. Moreover, Sarkisian et al. [10] designed a 12-story structure for the Administrative Office of the Courts. Life-cycle cost analysis assisted in informed decision making and system selection, and the final design featured a steel-framed superstructure with an isolation system. Chatzidaki [11] optimized the design of and economically evaluated reinforced-concrete (RC) isolated structures. Generally, the researchers have a similar conclusion: the life-cycle cost of isolated buildings is less than that of the fixed-base buildings.

In the current study, structural seismic performance is measured according to indicator-based life-cycle cost. This cost can synthesize all factors, including structural design, construction, and expected loss from earthquakes. The indicator of an isolated building is analyzed in detail in comparison with that of a fixed-base building in the following sections. Practical construction complexity, important but difficult to be included in initial cost analysis, is taken into due account by a proposed diversity index as another objective, this approximation data is best used for the preliminary design stage, and the large pools of alternatives leave to a design maker much freedom to select the one that best meets his/her goals.

## 2. Indicator-Based Life-Cycle Cost of Structural Seismic Performance

### 2.1. Life-Cycle Cost of Structures

The life-cycle cost of a structure may refer either to the design life of a new structure or to the remaining life of an existing or retrofitted structure. This cost can be expressed as a function of time and of the design vector [1]:

(1) $$\begin{array}{c} C_{\text{tot}}\left(t,s\right)=C_{\text{in}}\left(s\right)+e^{-\lambda t}C_{\text{ls}}\left(t,s\right), \end{array}$$

where *C*_tot_ is the total cost of a structure; *C*_in_ is the initial cost of a new or retrofitted structure; *C*_ls_ is expected loss; *t* is the time period; *s* is the design vector corresponding to the design loads, resistance, and material properties that influence the performance of the structural system; and *λ* is the constant annual discount rate and is usually equal to 3% [1].

*C*_ls_ can be written as [1]

(2) $$\begin{array}{c} C_{\text{ls}}\left(t,s\right)=\underset{j}{\sum }\;\underset{i}{\sum }[C_{\text{ls}}\left(B_{i}\right)P\left(B_{i}\;|\;I\right)]P\left(I_{j}\right), \end{array}$$

where *P*(*B*_*i*_∣*I*) is the conditional probability of failure, which can be obtained through dynamic reliability analysis. *I* is fortification intensity; *P*(*I*_*j*_) is the probability of seismic hazard; and *I*_*j*_ denotes the three seismic design levels, namely, minor (*I*_*s*_), moderate (*I*_*m*_), and major earthquakes (*I*_*l*_). The cumulative distribution function of fortification intensity is a type III extreme value distribution during the design reference period in Mainland China. Thus, *P*(*I*_*s*_), *P*(*I*_*m*_), and *P*(*I*_*l*_) are approximately equal to 70%, 25.2%, and 4.5%, respectively [12].

*B* represents structural damage and can be divided into five levels, that is, none, slight, moderate, severe, and collapsed. These structural damage states are defined by specific quantities. Interstory drift can be a reliable limit-state criterion according to which expected damage can be determined. Thus, maximum interstory drift (*θ*) is considered the response parameter that best characterizes structural damage. The damage index limits of isolated superstructures are similar to those of fixed-base buildings; however, these limits have not been determined for the isolated layer. The damage states are quantitatively defined in terms of interstory drift given that the isolator may be damaged when its shear strain exceeds the acceptable value as shown in Table 1.

*C*_ls_ corresponds to the limit-state economic and noneconomic loss from earthquakes. The economic loss consists of direct and indirect economic loss, relief costs, and long-term investment. Noneconomic loss mainly involves the loss of human life. The economic quantification of these losses depends on several socioeconomic parameters, and the most difficult cost to quantify is that which corresponds to the loss of human life. This loss can be estimated using various approaches that range from purely economic reasoning to more sensitive concepts that consider human loss to be irreplaceable. These cost components are related to RC building damage. *C* is initial building cost; *P* is the total number of human beings in the building; and *a*, *e*, and *d* are the ratios of indirect economic loss, relief cost, and structural content, respectively. These data are derived from [13], as shown in Table 2. Thus, *C*_ls_ can be determined based on initial cost and building function.

### 2.2. Indicator of Structural Seismic Performance

As with the coefficient of investment risk, the indicator of structural seismic performance can be expressed as

(3) φ=1−CinsCtott,s=1−CinsCins+e−λtClst,s=e−λtClst,sCtott,s,

where *φ* is the indicator of structural seismic performance based on life-cycle cost. It represents the proportion of expected damage to life-cycle cost. A high *φ* value increases the risk of the expected economic loss and indicates the poor seismic performance of a building.

## 3. Conditional Failure Probability of Isolated Buildings

The calculation of conditional failure probability is key to life-cycle cost evaluation. Thus, random dynamic analysis is also conducted in this study to determine dynamic reliability. Consequently, the conditional failure probability of isolated buildings can be calculated.

### 3.1. Random Dynamic Analysis of Isolated Buildings

The nonlinear behavior of isolated buildings is presented in a Bouc-Wen model. The equivalent linearization method is applied, and the equations of motion can thus be obtained:

(4) $$\begin{array}{c} M\ddot{Y}+C\dot{Y}+KY+K_{h}\upsilon =-ME{\ddot{u}}_{g},\\ \dot{\upsilon }+C_{eq}\dot{Y}+K_{eq}\upsilon =0, \end{array}$$

where

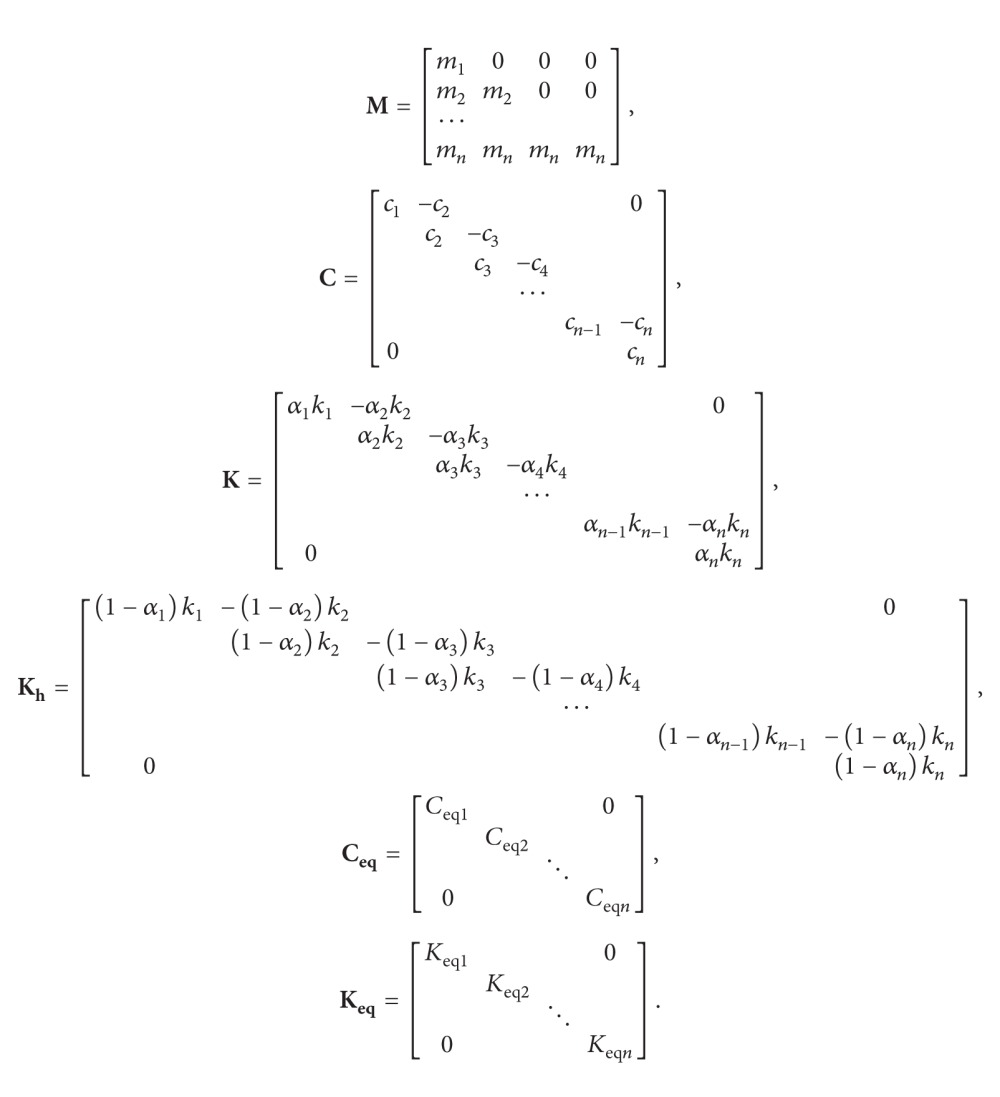
(5)
When the parameters of the Bouc-Wen model are *A* = 1, *n* = 2, then

(6) Ceqi=−1+2πβiσυi2ϕ−12sin2ϕ−π2+γiσυi2,Keqi=4πβiσx˙iσυiϕ−12sin2ϕ−π21−ρx˙iυi21.5      +ρx˙iυiϕ−12sin2ϕ−π2      +2γiEx˙iυi,ϕ=arctg1−ρx˙iυi2ρx˙iυi,  ρx˙izi=Ex˙iυiσx˙iσυi,

where **E** = {1 0 ⋯ 0}^*T*^, *c*_*i*_ = 2*m*_*i*_ *ωζ*_*i*_, and *ω* and *ζ*_*i*_ are the fundamental frequencies and the damping ratio of a building, respectively. Consider *ζ*_*i*_ = 0.05.

Assuming that the ground motion is stationary with zero mean and that the power spectral density of ground acceleration is expressed as the Kanai-Tajimi spectrum, the pseudoexcitation method can then be used to analyze the random response of a building.

Given pseudoexcitation ${\ddot{\tilde{u}}}_{g}^{}=\sqrt{S_{{\ddot{u}}_{g}}(\omega )}e^{i\omega t}$, the response of (4) can be written as

(7) $$\begin{array}{c} \tilde{Y}=Be^{i\omega t},\;\;\dot{\tilde{Y}}=i\omega Be^{i\omega t},\;\;\ddot{\tilde{Y}}=-\omega^{2}Be^{i\omega t},\\ \tilde{\upsilon }=B^{\prime }e^{i\omega t}, \end{array}$$

where **B** is the amplitude vector of the pseudoresponse. Consider

(8) $$\begin{array}{c} B=B_{r}+iB_{i}^{},\\ B_{r}=\frac{-G_{r}^{}}{G_{r}^{2}+G_{i}^{2}}E\sqrt{S_{{\ddot{u}}_{g}}\left(\omega \right)},\;\;B_{i}=\frac{G_{i}^{}}{G_{r}^{2}+G_{i}^{2}}E\sqrt{S_{{\ddot{u}}_{g}}\left(\omega \right)},\\ G_{r}=M^{-1}K-\omega^{2}I+\omega M^{-1}K_{h}T_{i}C_{eq},\\ G_{i}=\omega M^{-1}C-\omega M^{-1}K_{h}T_{r}C_{eq}, \end{array}$$

where **T**_**i**_ and **T**_**r**_ are the diagonal matrices. The respective diagonal elements are then obtained by

(9) $$\begin{array}{c} T_{i_{j}}=\frac{-\omega }{K_{\text{eq}j}^{2}+\omega^{2}},\;T_{r_{j}}=\frac{K_{\text{eq}j}^{}}{K_{\text{eq}j}^{2}+\omega^{2}},\;j=1,2,\ldots ,n,\\ B^{\prime }=\left(\omega T_{i}-i\omega T_{r}\right)C_{eq}B. \end{array}$$

If a set of *ω* can be generated, then the pseudoresponse can be determined. Finally, the power spectral density of the response is written as

(10) $$\begin{array}{c} S_{\dot{Y}}\left(\omega \right)={\dot{\tilde{Y}}}^{*}\cdot {\dot{\tilde{Y}}}^{T},\;\;S_{\upsilon }\left(\omega \right)={\tilde{\upsilon }}^{*}\cdot {\tilde{\upsilon }}^{T},\\ S_{\dot{Y}\upsilon }\left(\omega \right)={\dot{\tilde{Y}}}^{*}\cdot {\tilde{\upsilon }}^{T}. \end{array}$$

If the input is a stationary process with zero mean, then the response is a stationary process with zero mean as well. Thus,

(11) $$\begin{array}{c} \mu_{\dot{y}}=0,\;\;\sigma_{\dot{y}}^{2}=E\left[{\dot{y}}^{2}\right]=\overset{+\infty }{\underset{-\infty }{\int }}S_{\dot{Y}}\left(\omega \right)d\omega ,\\ \mu_{\upsilon }=0,\;\;\sigma_{\upsilon }^{2}=E\left[\upsilon^{2}\right]=\overset{+\infty }{\underset{-\infty }{\int }}S_{\upsilon }\left(\omega \right)d\omega ,\\ E\left[\dot{y}\upsilon \right]=\overset{+\infty }{\underset{-\infty }{\int }}S_{\dot{Y}\upsilon }\left(\omega \right)d\omega . \end{array}$$

The power spectral density of the structural response is computed using (4)~(11). Iteration may be required to produce a solution. We therefore obtain the mean square value of the displacement in each layer with

(12) $$\begin{array}{c} \sigma_{y}^{2}=E\left[y^{2}\right]=\overset{+\infty }{\underset{-\infty }{\int }}S_{Y}\left(\omega \right)d\omega . \end{array}$$

The mean, variance, and coefficient variation of the maximum interstory drift may be calculated using

(13) $$\begin{array}{c} \mu_{\theta_{mi}}=\frac{\mu_{y_{mi}}}{h_{i}},\;\;\sigma_{\theta_{mi}}=\frac{\sigma_{y_{mi}}}{h_{i}},\;\;\delta_{\theta_{mi}}=\frac{\sigma_{y_{mi}}}{\mu_{y_{mi}}}, \end{array}$$

where *h*_*i*_ is the height of each story and *μ*_*y*_*mi*__ and *σ*_*y*_*mi*__ are the mean and variance of the maximum displacement in each story, respectively. *μ*_*y*_*mi*__ and *σ*_*y*_*mi*__ can be written as [14]

(14) $$\begin{array}{c} \mu_{y_{mi}}=p_{i}\sigma_{y_{i}}^{},\;\;\sigma_{y_{mi}}^{}=f_{i}\sigma_{y_{i}}^{}. \end{array}$$

*p*_*i*_ and *f*_*i*_ are the factors of maximum earthquake acceleration and are expressed as

(15) $$\begin{array}{c} p_{i}=\sqrt{2\ln\left(\nu_{i}\tau \right)}+\frac{0.5772}{\sqrt{2\ln\left(\nu_{i}\tau \right)}},\;\;f_{i}=\frac{\pi }{\sqrt{12\ln\left(\nu_{i}\tau \right)}}. \end{array}$$

*ν*_*i*_ is the zero rate of earthquake response $\nu_{i}=\sigma_{{\dot{y}}_{i}}/\pi \sigma_{y_{i}}$. *τ* is the characteristic value of earthquake ground motion. *τ* = 5.5 for hard soil [12].

### 3.2. Conditional Probability of Failure in Isolated Buildings

The limit-state equation of isolated buildings can be expressed as

(16) $$\begin{array}{c} Z=\theta -\theta_{m}=0. \end{array}$$

The conditional failure probability is computed by

(17) $$\begin{array}{c} P_{f}=P\left(Z<0\right)=P\left(\theta <\theta_{m}\;|\;I=I_{0},\tau \right)=1-\Phi \left(\beta \right), \end{array}$$

where *I*_0_ and *β*_*θi*_ are the seismic fortification intensity and the reliability of the structure, respectively. The seismic response of the structure meets log-normal distribution if the structure is an RC frame. As per the JC method, *β*_*θi*_ is written as

(18) $$\begin{array}{c} \beta_{\theta i}=\frac{\ln\left[\mu_{\theta_{i}}\sqrt{1+\delta_{\theta_{mi}}^{2}}/\mu_{\theta_{mi}}\sqrt{1+\delta_{\theta_{i}}^{2}}\right]}{\sqrt{\ln\left(1+\delta_{\theta_{i}}^{2}\right)\left(1+\delta_{\theta_{mi}}^{2}\right)}}, \end{array}$$

where *μ*_*θ*_*i*__ is the mean value of the seismic isolation layer and *μ*_*θ*_*i*__ = *θ*_*i*_ and *δ*_*θ*_*i*__ is the variation coefficient of the maximum interstory drift ratio. *δ*_*θ*_0__ = 0.35 for the isolation layer and *δ*_*θ*_*i*__ = 0.3642 for the *i*th story. *μ*_*θ*_*mi*__ and *δ*_*θ*_*mi*__ can be computed with (13).

An isolated building fails if any floor fails; therefore, the reliability of this building can be determined using

(19) $$\begin{array}{c} \beta =\overset{n}{\underset{i=1}{\prod }}\beta_{\theta i}. \end{array}$$

## 4. Numerical Example and Analysis

The structural seismic performance of isolated buildings is compared with that of fixed-base buildings by taking the isolated building as a numerical example.

### 4.1. Numerical Example

A seven-story hospital building with a reinforced-concrete frame is designed for Gulang, China. The following design parameters are applied: seismic precautionary intensity 9; basic acceleration of ground motion 0.40 g; site class II; and total building area of 7789.51 m^2^.  Table 3 shows the other parameters for both isolated and fixed-base buildings. In addition, the parameters of the Bouc-Wen model for a superstructure are as follows:

(20) $$\begin{array}{c} A_{i}=1,\;\;n_{i}=2,\;\;\alpha_{i}=0.02. \end{array}$$

The parameter of the Bouc-Wen model for an isolated layer is

(21) $$\begin{array}{c} \alpha =0.1. \end{array}$$

### 4.2. Seismic Performance of Isolated Buildings Based on Life-Cycle Cost

#### 4.2.1. Initial Costs

The initial cost of the isolated building is 13.278 million yuan. Specifically, the isolators cost 810,000 yuan. This initial cost is higher than that of a fixed-base building because the seismic measures of the superstructure hospital building are strengthened by the requirements of Chinese seismic design provisions. The initial cost of a fixed-base building is reduced by 1% of that of an isolated building, that is, 13.145 million yuan, as per the statistical data [13].

#### 4.2.2. Expected Loss from Earthquakes

The building houses approximately 2,800 people.  Table 4 depicts the economic losses and the casualties under different situations according to the analysis above. *C* is the initial cost of the structure.

When the precautionary seismic intensity of the building is grade 9, the peak accelerations of the different earthquake levels, that is, minor, moderate, and strong earthquakes, are 1.4, 4.0, and 6.2 m/S^2^, respectively.  Table 5 displays the conditional probability of failures in the isolated and the fixed-base buildings at various earthquake-risk levels.

Assuming that the lifetime of the building is 50 years, the probabilities of exceeding this lifetime are 70%, 25.2%, and 4.5% given the varied earthquake levels.  Table 6 exhibits the expected loss at these levels. The total expected loss is expressed as the sum of the losses at the different risk levels; thus,

 the expected loss of the isolated building *C*_ls_ = 0 + 31.471 + 20.059 = 51.530 (10,000 yuan);
 the expected loss of the fixed-base building *C*_ls_ = 10.946 + 23.534 + 105.069 = 139.549 (10,000 yuan).

#### 4.2.3. Life-Cycle Costs

Life-cycle cost is the sum of the initial cost and the expected loss. Given the discounted factor over time *t* = 50 and annual constant discount rate *λ* = 0.03, then

 the life-cycle cost of the isolation building is *C*_tot_ = 1327.8 + 26.436*e* ^−0.03×50^ = 1333.7 (10,000 yuan);
 the life-cycle cost of the fixed-base building is *C*_tot_ = 1314.5 + 139.549*e* ^−0.03×50^ = 1345.6 (10,000 yuan).

#### 4.2.4. Indicator of Structural Seismic Performance

By substituting the initial and the life-cycle costs into (3), we can determine the seismic performance of the isolated and the fixed-base buildings.

Isolated building,

(22) $$\begin{array}{c} \varphi =1-\frac{C_{\text{in}}\left(s\right)}{C_{\text{tot}}\left(t,s\right)}=1-\frac{1327.8}{1333.7}=0.0044. \end{array}$$

Fixed-base building,

(23) $$\begin{array}{c} \varphi =1-\frac{C_{\text{in}}\left(s\right)}{C_{\text{tot}}\left(t,s\right)}=1-\frac{1314.5}{1345.6}=0.0231. \end{array}$$

The indicator of the structural seismic performance of the isolated building is much lower in value than that of the structural seismic performance of the fixed-base building. Therefore, isolated buildings are safer and less risky than fixed-base buildings.

## 5. Conclusion

This paper proposes an indicator of structural seismic performance based on life-cycle cost. The indicator is expressed as a ratio of lifetime damage loss to life-cycle cost. Thus, major factors are considered, including the uncertainty in hazard demand and structural capacity, nonlinear structural response behavior, balance of costs, and loss from earthquakes. Therefore, a high indicator value indicates the poor seismic performance of a building. We take an actual seven-story, isolated hospital building during an earthquake at Gulang, Gansu, China, as an example and conduct a random vibration analysis to determine the dynamic reliability and conditional failure probability. By substituting the dynamic reliability of the building, we evaluate the expected loss and life-cycle cost of the isolated building. The study conclusions can be summarized as follows.

1. The initial costs in the isolated case are higher by 1% than those in the corresponding fixed-base case. The sample building is a hospital; thus, the superstructure seismic measures are strengthened by the requirements of Chinese seismic design provisions.
2. Base isolation reduces conditional failure probability; therefore, the expected loss of the isolated building is only 37% of that of the fixed-base building. Moreover, the life-cycle cost of the isolated building decreases to nearly 1% of that of the fixed-base building. The base isolation reduces earthquake response and protects against such calamities. Hence, the isolated building effectively withstands future earthquakes.
3. The optimum design balances building reliability and building investment. The indicator based on life-cycle cost assists owners and engineers in making investment decisions in consideration of structural design, construction, and expected loss.

## Figures and Tables

**Table 1:** Damage index limits for RC frames.

Damage state		None	Slight	Moderate	Severe	Collapsed
Drift ratio	Superstructure	0.002	0.004	0.008	0.02	0.05
	Isolated layer	0.51	0.90	1.28	1.79	2.05

**Table 2:** Limit-state parameters for cost evaluation (10,000 yuan).

Damage state	None	Slight	Moderate	Severe	Collapsed
Economic loss	0	0.1(1 + *a* + *e*) × *C*	0.4(1 + *a* + *e*) × *C*	[0.7(1 + *a* + *e*) + 0.3*d*] × *C*	(1 + *a* + *e* + 0.95*d*) × *C*
Noneconomic loss	0	0.0011 × *P*	0.0268 × *P*	0.2050 × *P*	2.2350 × *P*

**Table 3:** Structural parameters of the numerical example.

	*k* (kN/m)		*β*		*γ*		Mass (10^3^ kg)	*h* (m)
	Isolation	Fixed-base	Isolation	Fixed-base	Isolation	Fixed-base		
Isolation layer	1513071	—	0.5	—	0.5	—	2142.9	0.3
1	1390000	1946000	3729	3729	−1243	−1243	2046.4	4.2
2	1470000	2058000	5302	5302	−1767	−1767	1957.2	3.3
3	1370000	1918000	6848	6848	−2283	−2283	1838.8	3.3
4	1290000	1806000	8237	8237	−2746	−2746	1488.7	3.3
5	1070000	1498000	11191	11191	−3730	−3730	878.8	3.3
6	1660000	2324000	93468	93468	−31156	−31156	435.4	1.5
7	204000	285600	30632	30632	−10211	−10211	135.4	3

**Table 4:** Economic and noneconomic losses in different damage states (10,000 yuan).

Damage state	None	Slight	Moderate	Severe	Collapsed
Economic loss	0	0.106 × *C*	0.424 × *C*	1.192 × *C*	2.485 × *C*
Noneconomic loss	0	3.08	75.04	574	6258

**Table 5:** Conditional probability of failure.

*B*_*i*_	Minor earthquake		Moderate earthquake		Strong earthquake	
	Seismic isolation	Not isolated	Seismic isolation	Not isolated	Seismic isolation	Not isolated
None	0.0489	0.0015	0.2710	1	0.4643	1
Slight	0	0.1098	0.8683	0.3427	0.9965	1
Moderate	0	0	0	0.0705	0.4740	1
Severe	0	0	0	0	0	0.7287
Collapsed	0	0	0	0	0	0

**Table 6:** Lifetime losses from damage in isolated and fixed-base buildings (10,000 yuan).

*B*_*i*_	Minor earthquake		Moderate earthquake		Major earthquake	
	Seismic isolation	Not isolated	Seismic isolation	Seismic isolation	Not isolated	Seismic isolation
None	0.000	0.000	0.000	0.000	0.000	0.000
Slight	0.000	10.946	31.471	12.299	6.450	6.409
Moderate	0.000	0.000	0.000	11.235	13.609	28.457
Severe	0.000	0.000	0.000	0.000	0.000	70.203
Collapsed	0.000	0.000	0.000	0.000	0.000	0.000

Total	0.000	10.946	31.471	23.534	20.059	105.069
